# Supplementary material for: MetaRibo-Seq measures translation in microbiomes
Source: Nat Commun. 2020 Jun 29;11:3268. doi: 10.1038/s41467-020-17081-z (PMC7324362; doi:10.1038/s41467-020-17081-z)
Supplement: Supplementary file 10 — Supplementary Data 7 [file 41467_2020_17081_MOESM10_ESM.zip › File2/Confidence_High_Taxonomy/238359_out.krona.html]

Javascript must be enabled to view this page.

members
magnitude
magnitudeUnassigned
count
unassigned
taxon
rank

238359\_out

3

superkingdom
3
2

976
1
phylum

1
class
200643

1
order
171549

family
1
815

816
genus
1

1263044

SRS1041132\_contig\_number\_1358
1
species

2
phylum
1239

1737404
class
1

order
1
1737405

1570339
family
1

543311
genus
1

species
1

SRS148284\_contig\_number\_contig-100\_7569.109762
33033

186801
1
class

186802
1
order

1
family
541000

genus
1
292632

2053618

SRS104084\_contig\_number\_448
1
species
